# BioPartsBuilder: a synthetic biology tool for combinatorial assembly of biological parts

**DOI:** 10.1093/bioinformatics/btv664

**Published:** 2015-11-14

**Authors:** Kun Yang, Giovanni Stracquadanio, Jingchuan Luo, Jef D. Boeke, Joel S. Bader

**Affiliations:** ^1^Department of Biomedical Engineering, Johns Hopkins University, 3400 N. Charles Street, Baltimore, MD 21218, USA and; ^2^Institute for Systems Genetics and Department of Biochemistry and Molecular Pharmacology, NYU Langone Medical Center, New York, NY 10016, USA

## Abstract

**Summary:** Combinatorial assembly of DNA elements is an efficient method for building large-scale synthetic pathways from standardized, reusable components. These methods are particularly useful because they enable assembly of multiple DNA fragments in one reaction, at the cost of requiring that each fragment satisfies design constraints. We developed BioPartsBuilder as a biologist-friendly web tool to design biological parts that are compatible with DNA combinatorial assembly methods, such as Golden Gate and related methods. It retrieves biological sequences, enforces compliance with assembly design standards and provides a fabrication plan for each fragment.

**Availability and implementation:** BioPartsBuilder is accessible at http://public.biopartsbuilder.org and an Amazon Web Services image is available from the AWS Market Place (AMI ID: ami-508acf38). Source code is released under the MIT license, and available for download at https://github.com/baderzone/biopartsbuilder.

**Contact:** joel.bader@jhu.edu

**Supplementary information:** Supplementary data are available at *Bioinformatics* online.

## 1 Introduction

DNA synthesis technologies are improving faster than Moore’s law, allowing the synthesis of genes, pathways (Ro *et* *al.*, 2006), bacterial genomes (Gibson *et* *al.,* 2010), eukaryotic chromosomes (Annaluru *et* *al.,* 2014; Dymond *et al.,* 2011) and eventually entire eukaryotic genomes. Many projects have individual ‘parts’ as synthetic targets, such as promoters, coding domains and transcriptional terminators. While individual parts can be characterized, predicting how parts will operate together remains a challenge. Rather than building a single construct, therefore, it can be more efficient to specify multiple alternatives for each part, then use massively parallel synthesis and assembly to generate a combinatorial library that can be screened for the desired function. In particular, Golden Gate assembly is an efficient and effective strategy to assemble combinatorial libraries (Engler *et* *al.*, 2009). However, Golden Gate assembly requires a computationally challenging design step to create ‘standardized’ parts that have compatible overhangs, lack pre-defined restriction sites and comply with other constraints.

To streamline the process of designing standardized biological parts for Golden Gate assembly, we developed BioPartsBuilder, which retrieves sequence data from different sources and ensures compliance with design standards that are compatible with combinatorial assembly. Though there are tools for automated parts retrieval (Scher *et* *al.*, 2014) and subsequent primer design for DNA assembly (Bode *et* *al.*, 2009; Rouillard *et* *al.,* 2004), the choices for Golden Gate assembly are limited. Compared with existing Golden Gate designers (Hillson *et* *al.*, 2012), BioPartsBuilder is distributed open source software and freely modified by both academic and commercial users. BioPartsBuilder also provides a repository system that stores the designed parts and shares data within members associated with the same laboratory. BioPartsBuilder therefore provides useful, new, integrated and extendable functionality for the synthetic biology community.

## 2 Software modules

**Fig. 1.**
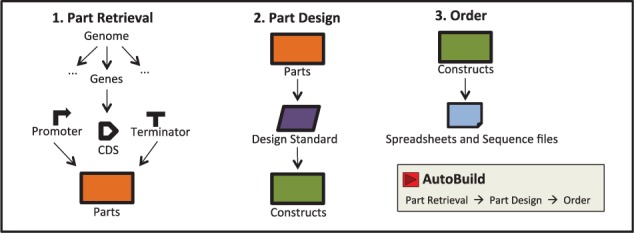
BioPartsBuilder provides easy interface to retrieve, design and order parts. An AutoBuild module is available for one-click design

BioPartsBuilder provides an easy interface to retrieve, design and order parts (Fig. 1) that are compatible with Golden Gate (Engler *et* *al.*, 2009), BglBrick (Anderson *et* *al.*, 2010) or user-defined assembly standards.

### 2.1 Part retrieval

BioPartsBuilder implements a sophisticated sequence retrieval system to gather data from different sources. Users can submit a list of RefSeq protein/nucleotide accession numbers to retrieve sequences and annotations from NCBI, or for parts without RefSeq accessions or with customized sequences and annotations, users can upload a file in Fasta or CSV format. As retrieving a large number of arbitrary parts from a genome and upload to the system is tedious, BioPartsBuilder implements an advanced search engine for retrieving parts from annotated genomes, similar to G enomeCarver software (Scher *et* *al.*, 2014). It parses annotations, generates and stores a search index, and provides access to structured search terms (Supplementary Tables S1 and S2) through the Apache SOLR query language.

### 2.2 Part design

Parts imported into BioPartsBuilder can be re-designed according to pre-defined or additional user-defined design standards. Users can customize the design workflow to perform one or more of the following steps.

- Codon optimization: Users can specify host organism for codon optimization. This option can be left blank for parts that lack protein–coding regions, such as promoters or terminators. This step can be amended also to accommodate vendors’-specific recoding strategies.
- Restriction enzyme constraints: Combinatorial assembly techniques largely rely on the use of specific restriction enzymes to create a unique assembly. For this reason, BioPartsBuilder provides: (a) restriction enzyme remover that changes the nucleotide sequence to avoid restriction sites corresponding to a user-specified list of restriction enzymes; and (b) restriction enzyme locator that detects the presence of user-specified restriction enzymes without recoding the sequence.
- Prefix and suffix insertion: Users can specify sequences to be added to the beginning and the end of each part.
- Fabrication: Parts can be larger that the synthesis capability of commercial providers. In this case, BioPartsBuilder splits the sequence in fragments of user-defined length using unique overlaps, which allow unambiguous assembly (see Supplementary Text).

BioPartsBuilder assigns a unique Job ID to each design task. Users can check the status and error report of design tasks online. When the design task is finished, BioPartsBuilder sends an email to notify the user.

### 2.3 Order

Design results are accessible online. And users can also use the Order module to collect specific designs and prepare files for ordering parts from companies. The Order module creates statistical summaries for user-selected designs and provides tables of parts, constructs and design standards. It generates spreadsheets, sequence files and summary report files for users to download.

### 2.4 AutoBuild

To streamline the entire design process, BioPartsBuilder has a fully automated design module, AutoBuild, which allows users to retrieve, design and create orders for a batch of parts with one click. This module serves as a convenient ‘wizard’ for users whose needs are met by the most common design standards, which are already defined in the software.

### 2.5 Data storage and administration

BioPartsBuilder organizes data and users by laboratories. People in the same laboratory can share parts and designs that remain private to other laboratories and the public. BioPartsBuilder provides an administration panel specifically for laboratory administrators to manage members and design standards. The initial creator of a new laboratory in BioPartsBuilder automatically becomes the laboratory administrator.

## 3 Design workflow example

Using Autobuild it is possible to quickly design both coding and non-coding parts for Golden Gate using these two workflows.

### 3.1 Coding region

In the ‘Search Genomes’ tab, input query ‘systematic name:YBR019C’; select ‘Golden Gate – CDS’ design standard and assign a name to the Order. Click create parts, then select the part and confirm your design.

#### 3.2 Non-coding region

In the ‘Search Genomes’ tab, input query ‘systematic name:YBR019C promoter’; select ‘Golden Gate – NonCDS’ design standard and assign a name to the Order. Click create parts, then select the part and confirm your design.

## Supplementary Material

Supplementary Data
