# Supplementary material for: BioPartsBuilder: a synthetic biology tool for combinatorial assembly of biological parts
Source: Bioinformatics. 2015 Nov 14;32(6):937–9. doi: 10.1093/bioinformatics/btv664 (PMC4803390; doi:10.1093/bioinformatics/btv664)
Supplement: Supplementary Data [file supp_32_6_937__index.html]

BioPartsBuilder: a synthetic biology tool for combinatorial assembly of biological parts — BioPartsBuilder: a synthetic biology tool for combinatorial assembly of biological parts — BioPartsBuilder: a synthetic biology tool for combinatorial assembly of biological parts — Supplementary Data 

# BioPartsBuilder: a synthetic biology tool for combinatorial assembly of biological parts

## Supplementary Data

files

- Supplementary Data - pdf file
