# Supplementary material for: BioPartsBuilder: a synthetic biology tool for combinatorial assembly of biological parts
Source: Bioinformatics. 2015 Nov 14;32(6):937–9. doi: 10.1093/bioinformatics/btv664 (PMC4803390; doi:10.1093/bioinformatics/btv664)
Supplement: Supplementary Data [file supp_btv664_supplementary.pdf]

Supplementary Text
BioPartsBuilder: a synthetic biology tool for combinatorial assembly of biological parts

Kun Yang[1], Giovanni Stracquadanio[1], Jingchuan Luo[2], Jef D. Boeke [2], and Joel S. Bader[1]
[1] *Department of Biomedical Engineering and High-Throughput Biology Center, Johns Hopkins University, Baltimore, MD 21218, USA*
[2] *Institute for Systems Genetics and Department of Biochemistry and Molecular Pharmacology, NYU Langone Medical Center, New York, NY 10016, USA*

PART RETRIEVAL USING THE APACHE SOLR QUERY LANGUAGE

Retrieving a large number of arbitrary parts from a genome and upload to the system is tedious. For this reason, BioPartsBuilder implements an advanced search engine for retrieving parts from annotated genomes through the Apache SOLR query language (`http://lucene.apache.org/solr`). Table S1 lists structured search terms and Table S2 provides examples of advanced queries.

TABLE S1: Fields for annotated genome queries

**Field**	**Description**	**SOLR Query Example**
systematic_name	The systematic name used in the genome annotation	systematic_name:YAL*
gene_name	Official gene symbol	gene_name:SEO1
organism	Organism latin name	organism:Saccharomyces cerevisiae
chromosome	Chromosome name	chromosome:chrII
feature	Entity type, including CDS, tRNA, telomere, promoter[1], terminator[2]	feature:CDS
start	The start position on chromosomes	start:[1000 TO *]
end	The end position on chromosomes	end:7235
strand	Strand, W or C (W: Watson, C: Crick)	strand:W
orf_classification	ORF classification: verified, dubious, uncharacterized, ...	orf_classification:verified
ontology_term	ontology accession number	ontology_term:0008150
description	Gene description	description:shock protein

[1]The promoter region is defined as 500 bp upstream of CDS or till gene boundaries
[2]The terminator region is defined as 100 bp downstream of CDS or till gene boundaries

TABLE S2: Advanced SOLR Query Examples

**SOLR Query**	**Description**
feature:CDS AND start:[1 TO 1000] AND strand:W	Retrieve CDS in the positive strand with start position in the first 1000 bp of all the chromosomes across all the organisms in the database
feature:tRNA AND chromosome:chrIII AND organism:“Saccharomyces cerevisiae”	Retrieve all the tRNA of yeast chromosome III
description:“shock protein” AND organism:“Saccharomyces cerevisiae”	Retrieve all the shock protein related entries of yeast
feature:gene AND ontology_term:0008150 AND orf_classification:verified AND chromosome:chrIII	Retrieve verified genes of chromosome III that are involved in biological process

PART FABRICATION

BioPartsBuilder provides a convenient function for users to fabricate parts that are larger than can be conveniently synthesized by commercial providers. For example, Gen9 Genebits are limited to 1 Kb in length. If a desired CDS or other synthesis target is 2.5 Kb, 3 Genebits will be required. The crucial design step is to ensure that three DNA fragments can be assembled correctly to obtain the expected sequence.

To use this assembly approach, we designed a greedy-algorithm that breaks the input sequence into fragments, which can be assembled unambiguously. Users specify the maximum fragment length ($L_{\max}$), the internal prefix and suffix (F_p, F_s), and the size of overlap (L_o) or a set of admissible overlap sequences (S_o). For a sequence of total length L bp longer than a vendor maximum sequence length θ bp, BioPartsBuilder will split the sequence into fragments as follows:

1. *Sequence segmentation.* The algorithm calculates the optimal number of fragments for the part as $n = \lceil L/L_{\max} \rceil$, and it splits the sequence into n fragments.
2. *Overlap encoding*: introduce L_o bp overlap between fragments. The overlap between segments i and $i+1$ consists of the last $\frac{L_o}{2}$ bp of fragment i and the first $\frac{L_o}{2}$ bp of fragment $i+1$.
   If the overlap is not unique or not in the user-defined overlap list, the algorithm iteratively shifts the split position between fragments, until an admissible overlap is found.
   If no valid overlap is found, the algorithm stops with an error.
3. *Standardization*: add an standard prefix and suffix to the first and last fragment of the part.

Encoding overlaps is the crucial step of the fabrication process. The overlap encoding procedure relies on perfect matching only; while it could represent an issue for long overlaps, it is generally reasonable when dealing with short overlaps, which is the case for fragment assembly. However, the algorithm is flexible enough to be extended with different strategies, such as imperfect matching or secondary structure predictions.

IMPLEMENTATION

The software is implemented using Ruby-on-Rails (`http://rubyonrails.org`) open-source framework (v3.2.11) and the MySQL database server. The web interface is designed with the Twitter Bootstrap framework (`http://getbootstrap.com`) for compatibility with standard computers and mobile devices.

BioPartsBuilder uses the BioRuby library to retrieve sequences and annotations [2]. The software uses ElasticSearch (`http://www.elasticsearch.org`) to perform search operations on annotated genomes. The public BioPartsBuilder website (`http://public.biopartsbuilder.org`) has *Saccharomyces cerevisiae* and *Escherichia coli* in the system. Users can add genomes to the stand alone version by using rake tasks. The command '`bundle exec rake partsBuilder:gff:import`' will retrieve all the annotations from a GFF3 file and import the data to the database. The command '`bundle exec rake partsBuilder:promoter_terminator:create`' will create annotations for promoters and terminators. The promoter and terminator are defined as the 500 bp upstream and 100 bp downstream of CDS or till gene boundaries, respectively. Users can change the definition of promoter and terminator by modifying the corresponding rake task. To add annotations to the ElasticSearch index, users can execute the command '`bundle exec rake partsBuilder:tire:import_annotation`'.

BioPartsBuilder uses GeneDesign modules [3] for codon optimization and restriction sites recoding. The restriction enzyme tables are inherited from GeneDesign and include 3689 commercially available restriction enzymes. The codon usage tables are also inherited from GeneDesign and currently include *Bacillus subtilis*, *Caenorhabditis elegans*, *Drosophila melanogaster*, *Escherichia coli*, *Homo sapiens*, and *Saccharomyces cerevisiae.* GeneDesign permits import of custom codon usage tables beyond these standard tables [3].

Since the design tasks can be computationally expensive, BioPartsBuilder uses a queue system to process jobs in batch and increase parallelism; the current implementation relies on Sidekiq (`https://github.com/mperham/sidekiq`), a Redis-backed Ruby library.

BioPartsBuilder can be deployed on any Unix environment. Detailed instructions are available on the wiki `https://github.com/baderzone/biopartsbuilder/wiki/Installation`.

[1] Agmon, N., Mitchell, L. A., Cai, Y., Ikushima, S., Chuang, J., Zheng, A., Choi, W.-J., Martin, J. A., Caravelli, K., Stracquadanio, G., and Boeke, J. D. (2015). Yeast golden gate (ygg) for the efficient assembly of s. cerevisiae transcription units. *ACS synthetic biology*.

[2] Goto, N., Prins, P., Nakao, M., Bonnal, R., Aerts, J., and Katayama, T. (2010). Bioruby: bioinformatics software for the ruby programming language. *Bioinformatics*, **26**(20), 2617–2619.

[3] Richardson, S. M., Nunley, P. W., Yarrington, R. M., Boeke, J. D., and Bader, J. S. (2010). Genedesign 3.0 is an updated synthetic biology toolkit. *Nucleic acids research*, **38**(8), 2603–2606.
